# Social Determinants in Clinical Decision Making: A Case of Mistaken Hepatic Encephalopathy

**DOI:** 10.7759/cureus.40405

**Published:** 2023-06-14

**Authors:** Mohamad K Almujarkesh, Anirudh R Damughatla, Asmita Shukla, Saqr Alsakarneh, Pragna Koleti, Dana LaBuda, Diane L Levine

**Affiliations:** 1 Internal Medicine, Wayne State University Detroit Medical Center, Detroit, USA; 2 Internal Medicine, University of Missouri Kansas City, Kansas City, USA; 3 Internal Medicine, Suburban Community Hospital, Norristown, USA; 4 Internal Medicine, Wayne State University, Detroit, USA

**Keywords:** hepatic encephalopathy, physician bias, specialty bias, implicit bias, social determinants in clinical decision making

## Abstract

Implicit (i.e., unconscious) bias frequently differs from one's explicit or conscious convictions. As humans, we rely on information and experiences that are repeatedly reinforced until they become reflexive, shaping our perceptions of reality. Specialty bias, a form of implicit bias specific to an individual's medical specialty, is a form of this bias. These cognitive processes of making assumptions aid efficient decision-making and likely confers an evolutionary advantage. However, automatic thinking can contribute to stereotyping, prejudice, and discrimination at both explicit and implicit levels. Despite a person's explicit beliefs evolving, the lasting implicit bias significantly impacts their behavioral interactions with individuals from stereotyped groups.

We present a case of an 83-year-old non-English speaking gentleman with a reported past medical history of an ischemic stroke who presented with acute encephalopathy and fever without jaundice and Aspartate transaminase/ Alanine transaminase (AST/ALT) of 64 and 34, respectively. He was initially treated for acute meningoencephalitis in the Neurologic Intensive Care Unit. With no clinical improvement in symptoms, his care was transferred to the Internal Medicine service later that week, and it was noted that he had features consistent with liver disease. Further history-taking revealed that the patient was intermittently confused with episodes of constipation. On examination, he had palmar erythema and asterixis, and additional labs showed elevated liver enzymes and ammonia levels. Computerized Tomography of the abdomen was suggestive of cirrhosis. He was treated for hepatic encephalopathy with lactulose and rifampin, with improvement in his mental status.

We believe our patient's clinical diagnosis was compromised by incomplete information related to a language barrier, and anchoring biases prevented a thorough history taking from the patient family and later on from the patient. Physician's anchoring bias, a form of implicit bias, can negatively impact outcomes in patients, especially those with limited language proficiency, due to communication barriers leading to misunderstanding of the patient's clinical presentation and overreliance on clinical heuristics.

## Introduction

Biases, stereotyping, prejudice, and discrimination affect the socioeconomic inequality between gender, race, and ethnic groups in America [1-2]. These biases and stereotypes have made their way into healthcare practices influencing physicians' clinical decisions via implicit bias. Unlike explicit prejudices, implicit bias is unconscious and at odds with one's beliefs and behaviors [1-2]. These implicit biases stem from stereotypes, heuristics, and disparities that exist in our society surrounding socioeconomic status, gender, race, sexuality, access to healthcare, and education level. Research studies have demonstrated that implicit biases play a role in healthcare disparities by subconsciously impacting clinical decisions and resulting in unequal medical treatment based on race, ethnicity, gender, or other characteristics [3-5]. It has been shown that even when there are comparable conditions of income, age, and insurance, there is still a significant disparity between racial and ethnic groups in healthcare outcomes and quality of care [3-5]. Disparities in treatment, prevention, and outcomes appear more significant when physicians recommend a test or referral for a procedure or drug and lower when they perform emergency surgery [5-8]. Preventative care shows the most significant difference in terms of disparities based on race and ethnicity, correlating with the increasingly worse presentations of patients of minority groups [6]. When considering implicit biases, physicians need to be aware of them and the heuristic shortcuts used in practice, as they lead to significant errors, disparities, and higher costs in the healthcare system [7].

## Case presentation

An 83-year-old Arabic-speaking gentleman with a reported prior medical history of ischemic stroke presented to the emergency department with a medical code with a 2-day history of worsening confusion, lethargy, and generalized weakness. As a result, his son called 911, and the patient was brought to the ED. On presentation, the patient was found to be hypertensive at 185/80 mmHg, tachycardic at 102 BPM, tachypneic at 24 breaths/minute, febrile at 38.5°C, saturating 98% on 2 liters nasal cannula.

On Initial examination by the ED team, the patient was poorly responsive, speaking in monosyllables but in Arabic, lethargic, and had a Glasgow Coma Score of 10. The preliminary information, which detailed his acute alterations in mental status, was provided to the ED team by the patient's son, who was also not fluent in English; ED staff did document this history in the EMR. The patient was negative for meningeal signs, including, Kernig's, Brudzinski's, and nuchal rigidity. Moreover, there was no documentation of tremors, hepatomegaly, or ascites on the Initial history and physical. Initial laboratory tests showed a hemoglobin level of 13.3 g/mL, hematocrit of 34.8%, white blood cell (WBC) count of 8.4, platelets 142,000; aspartate transaminase (AST): 64 IU/Liter, alanine transaminase (ALT): 34 IU/Liter, alkaline phosphatase (ALP): 288 IU/Liter, total bilirubin: 1.30 mg/dL, albumin 3.1 g/dL, Prothrombin time (PT): 12.0s, international normalized ratio (INR): 1.13, partial thromboplastin time (PTT): 28s, Lactic acid of 2.5. Due to concerns about airway protection patient was intubated and mechanically ventilated. The alcohol level was undetectable; the Urine drug screen was negative. Blood culture showed gram-positive cocci in clusters in the aerobic bottle.

**Figure 1:**
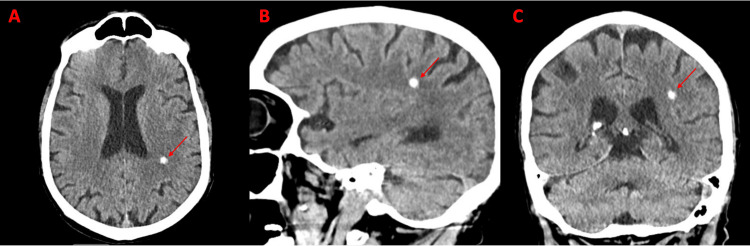
CT Head without contrast findings suggest a small focus of intraparenchymal hemorrhage involving the left frontoparietal centrum semiovale (Shown by red arrows). A: Axial View, B: Sagittal View, C: Coronal View.

**Figure 2:**
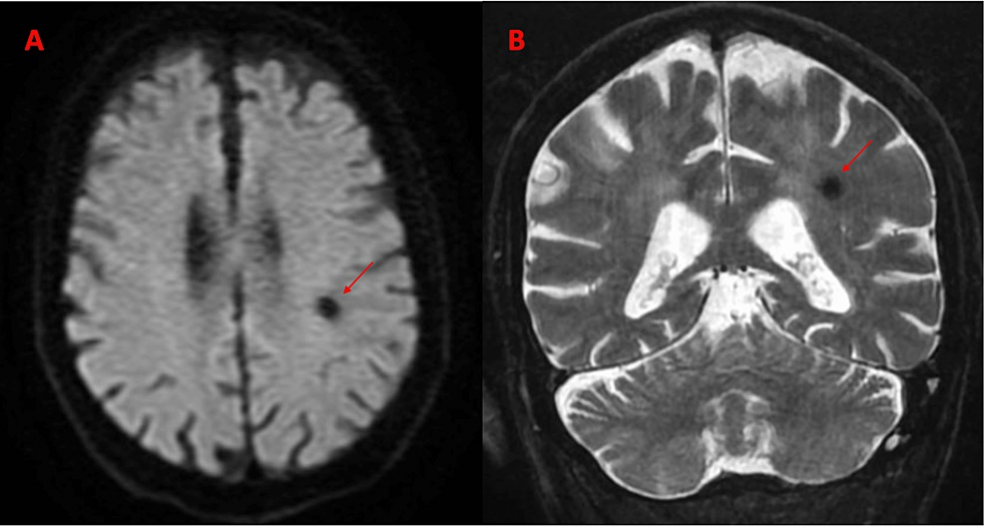
MRI with and without Contrast findings suggest no acute ischemic infarct but a focal 8 mm T2 hypointense lesion in the left posterior frontal lobe representing a calcified hematoma (shown by red arrows). It also showed nonspecific multiple T2/FLAIR hyperintense foci in the supratentorial white matter, likely due to chronic small vessel ischemia. A: Axial Diffusion-weighted imaging, B: Coronal T2 Image.

CT of the head without contrast showed a hyperdense lesion with suspected bleeding (Figure 1). MRI with and without contrast was done, which ruled out ischemic stroke, and findings suggest a small, calcified lesion representing a small stable hematoma (as shown in Figure 2). NICU was consulted, and the patient was admitted to the Neurology Intensive Care Unit (NICU), managed by a neurocritical care physician, for suspicion of intracranial bleeding, Sepsis with Acute meningitis/bacteremia, and hypertensive emergency. At this time, there was no mention or documentation of the team obtaining a detailed history from the son or family, and NICU pursued further workup regarding the stroke.

Lumbar puncture was attempted twice but was unsuccessful, at which point IR was consulted. Nevertheless, considering the patient's encephalopathic condition and bacteremia with Gram-positive cocci, it was justifiable to start empirical treatment for acute meningitis with a regimen of vancomycin, ceftriaxone, and ampicillin. On day two, the patient self-extubated, and on day three, given that stroke was less likely, the patient was transferred to the Internal Medicine service; by this time, the family was available at the bedside. 

On day three, at the time of transfer to the medical floors, in the presence of the family, the patient was alert and oriented x 1 to 2, and the family noted that the patient does not speak or understand English. Moreover, despite the appropriate treatment for meningitis and sepsis, the patient showed no significant improvement, leading to re-evaluating the original diagnosis. At this time, interpretation services were used to obtain as much history from the patient as possible (which was not documented to be done before), as he was only Arabic speaking. Further discussions revealed that thorough history-taking was never done with the son since English was not his first language. He was more comfortable and able to give more information when the conversation was carried out in Arabic with the help of interpreting services. This was the first time any team had spoken to the family since the initial encounter with ED. Upon further questioning, it was revealed that the patient had frequent episodes of constipation coinciding with brief periods of confusion. The son also noted that his confusion would improve, and he would return to baseline with the resolution of his constipation. A focused physical examination showed asterixis and palmar erythema suggestive of liver disease, raising the suspicion of hepatic encephalopathy, but no ascites were noted. At this time, Ammonia level was also obtained, elevated at 244 Micromole/L. 

Based on the above information, the medical team reviewed laboratory data comprehensively since admission, demonstrating elevated Liver function tests (LFTs), low albumin, prolonged PT and PTT, and elevated ammonia. By this time, cerebrospinal fluid (CSF) and repeat blood cultures were negative. The patient was then treated for hepatic encephalopathy with lactulose and rifaximin. After starting this regimen, his mental status significantly improved and returned to baseline over the next few days. An abdominal CT scan was obtained following his improvement in mentation, which revealed liver cirrhosis and signs of esophageal and rectal varices. Esophagogastroduodenoscopy (EGD) and colonoscopy were done, which revealed four large esophageal varices requiring band ligation and very large rectal varices. Viral studies for hepatitis B and C were negative. The patient, however, was found to have elevated anti-smooth muscle antibody titer, specifically anti-actin antibodies with an elevated IgG, suggestive of possible autoimmune hepatitis etiology. The patient was discharged in improved condition, with further workup planned as an outpatient.

## Discussion

Throughout history, humans have exhibited implicit bias, and as physicians, we are not exempt from its effects. Moreover, situations involving limited information and time constraints may encourage using stereotypes and heuristics to facilitate quick decision-making [4-7]. Furthermore, medical training often focuses on group-level data, such as population risk factors, and may even expose trainees to marginalized groups in unfavorable situations of illness or addiction, perpetuating negative stereotypes [3,7].

In the United States, physicians, especially white physicians, display these biases in clinical practice [4]. One study found that white physicians are more likely to perceive minority patients as less trustworthy, more contentious, and less likely to adhere to treatment recommendations than white patients [8]. At the implicit level, physicians prefer white over black patients, seeing white patients as more compliant and cooperative [8]. Higher physician implicit bias was associated with greater physician verbal dominance, which they defined as a more significant number of definitive physician statements relative to patient statements [8].

While race is a significant area where physicians exhibit implicit bias, it is not the only one; In our case, we would like to highlight another bias toward non-native non-English speaking patients. Patients who do not speak English typically experience more healthcare disparities, resulting in various issues, such as the absence of a personal primary care provider, inadequate oral health, increased rates of obesity, speech communication difficulties, and a lack of health insurance [9]. Numerous studies found that individuals who speak with an accent because their first language is something other than or do not speak English, are judged with negative stereotypes [10-13]. Language barriers and stereotypes within this population can result in unfavorable medical outcomes, insufficient adherence to treatment, and limited comprehension of medical conditions [9].

Another critical bias to take into consideration, in this case, is the specialty bias. A bias, not limited to medicine, is seeing what we are trained to find. Physicians also tend to overwhelmingly recommend treatments that have more knowledge about and have more training to deliver [14-15]. This type of bias is unavoidable in the practice of medicine. As in this case, when the patient was first admitted to the neurocritical care service, their differential diagnosis and treatment focused on having a neurologic etiology to explain his presentation. This allowed his clinical presentation and lab findings suggestive of a cirrhotic etiology to be overlooked, as, with the anchoring effect, physicians tend to support our initial diagnosis even with conflicting information present. After initial contact with the son (who is English-speaking) by the ED team, there appears to be no documentation of the NICU team further pursuing to obtain more information from the family regarding the patient's past medical history and baseline condition at home.

Given the high incidence of circumstances where behaviors might represent bias in healthcare, receiving feedback related to bias is essential. As a result, physicians are likely to attempt to self-regulate these potentially harmful behaviors [14]. Nevertheless, literature shows that people's ability to self-regulate their behaviors depends on how aware they are of their racial prejudice and how difficult the behaviors are to self-regulate [14]. Specifically, people are better at self-regulating explicit racial bias manifested in planned behaviors, such as verbal behaviors, than regulating implicit racial bias manifested in spontaneous behaviors, such as nonverbal and paraverbal behaviors [14]. Thus, even if non-Black physicians can successfully self-regulate negative explicit racial bias manifested in more planned behaviors, their implicit racial bias may still be manifested in spontaneous behaviors that are more difficult to self-regulate [14].

Demonstrating physicians have measurable implicit and specialty bias does not prove that bias affects patient-doctor interactions or alters the treatment patients receive [2,3]. However, research supports a relationship between patient care and physician bias in ways that perpetuate healthcare disparities, and literature supports a link between treatment decisions and implicit provider bias [2,3]. Given the growing evidence that implicit bias in physician decision-making contributes significantly to perpetuating healthcare disparities, it is crucial to find ways to reduce its impact [3-7].

One effective strategy for reducing implicit bias is through individuating; Individuation involves a conscious effort to focus on specific information about an individual, making it more salient in decision-making than that person's social information (e.g., race or gender) [2,3]. This is what occurred when our patient's care was transferred to medicine. The medicine team looked at the patient via individuating and obtained more precise information from his family, aiding in the diagnosis. Unfortunately, medicine is often practiced under time-pressured circumstances leading to overlooked errors and mistakes that affect patient care and outcomes [1-3]. Perspective-taking is another strategy to mitigate the impact of implicit bias. Perspective-taking is a conscious attempt to envision another person's viewpoint, reducing implicit bias in social interactions [3,10]. Undoubtedly, social divisions extend beyond just race, gender, age, or weight, and the effects of implicit social bias are likely more intricate than what data can convey [2]. Nevertheless, individuating and perspective-taking can help reduce bias and be effective regardless of the patient's social category [2].

## Conclusions

Our cases show physicians' implicit and anchoring biases can negatively impact patient outcomes. It is pivotal that physicians should be aware of these biases and heuristic shortcuts that are used in practice because they lead to significant errors, disparities, and higher costs in the healthcare system. Moreover, as physicians, we should be conscious of our biases and use strategies such as perspective-taking and individuating to reduce biases in daily practice.
